# Inhibition of *Acinetobacter baumannii* Biofilm Formation Using Different Treatments of Silica Nanoparticles

**DOI:** 10.3390/antibiotics12091365

**Published:** 2023-08-24

**Authors:** Iyad Y. Natsheh, Mallak T. Elkhader, Ala’a A. Al-Bakheit, Majd M. Alsaleh, Bassam I. El-Eswed, Nedaa F. Hosein, Duaa K. Albadawi

**Affiliations:** 1 Department of Medical Applied Sciences, Zarqa University College, Al-Balqa Applied University, Salt 19117, Jordan; iyadnatsheh@bau.edu.jo (I.Y.N.); malakelkhader95@gmail.com (M.T.E.); nedaamicrobiology@bau.edu.jo (N.F.H.); duaakhaledalbadawi@gmail.com (D.K.A.); 2 Department of Nutrition and Food Processing, Faculty of Agricultural Technology, Al-Balqa Applied University, Salt 19117, Jordan; alaa.al-bakheit@bau.edu.jo; 3 Department of Biology, School of Science, University of Jordan, Amman 11942, Jordan; 4 Department of Basic Science, Zarqa University College, Al-Balqa Applied University, Salt 19117, Jordan; bassameswed@bau.edu.jo

**Keywords:** ESKAPE, quorum sensing, opportunistic, nosocomial pathogen, multi-drug resistance, planktonic form

## Abstract

There exists a multitude of pathogens that pose a threat to human and public healthcare, collectively referred to as ESKAPE pathogens. These pathogens are capable of producing biofilm, which proves to be quite resistant to elimination. Strains of *A. baumannii*, identified by the “A” in the acronym ESKAPE, exhibit significant resistance to amoxicillin in vivo due to their ability to form biofilm. This study aims to inhibit bacterial biofilm formation, evaluate novel silica nanoparticles’ effectiveness in inhibiting biofilm, and compare their effectiveness. Amoxicillin was utilized as a positive control, with a concentration exceeding twice that when combined with silica NPs. Treatments included pure silica NPs, silica NPs modified with copper oxide (CuO.SiO_2_), sodium hydroxide (NaOH.SiO_2_), and phosphoric acid (H_3_PO_4_.SiO_2_). The characterization of NPs was conducted using scanning electron microscopy (SEM), while safety testing against normal fibroblast cells was employed by MTT assay. The microtiter plate biofilm formation assay was utilized to construct biofilm, with evaluations conducted using three broth media types: brain heart infusion (BHI) with 2% glucose and 2% sucrose, Loria broth (LB) with and without glucose and sucrose, and Dulbecco’s modified eagle medium/nutrient (DMEN/M). Concentrations ranging from 1.0 mg/mL to 0.06 µg/mL were tested using a microdilution assay. Results from SEM showed that pure silica NPs were mesoporous, but in the amorphous shape of the CuO and NaOH treatments, these pores were disrupted, while H_3_PO_4_ was composed of sheets. Silica NPs were able to target *Acinetobacter* biofilms without harming normal cells, with viability rates ranging from 61–73%. The best biofilm formation was achieved using a BHI medium with sugar supplementation, with an absorbance value of 0.35. Biofilms treated with 5.0 mg/mL of amoxicillin as a positive control alongside 1.0 mg/mL of each of the four silica treatments in isolation, resulting in the inhibition of absorbance values of 0.04, 0.13, 0.07, 0.09, and 0.08, for SiO_2_, CuO.SiO_2_, NaOH.SiO_2_ and H_3_PO_4_.SiO_2_, respectively. When amoxicillin was combined, inhibition increased from 0.3 to 0.04; NaOH with amoxicillin resulted in the lowest minimum biofilm inhibitory concentration (MBIC), 0.25 µg/mL, compared to all treatments and amoxicillin, whereas pure silica and composite had the highest MBIC, even when combined with amoxicillin, compared to all treatments, but performed better than that of the amoxicillin alone which gave the MBIC at 625 µg/mL. The absorbance values of MBIC of each treatment showed no significant differences in relation to amoxicillin absorbance value and relation to each other. Our study showed that smaller amoxicillin doses combined with the novel silica nanoparticles may reduce toxic side effects and inhibit biofilm formation, making them viable alternatives to high-concentration dosages. Further investigation is needed to evaluate in vivo activity.

## 1. Introduction

There exist several pathogens that instill fear in humanity and pose significant risks to public healthcare, including but not limited to *Enterococcus* spp., *Staphylococcus aureus*, *Klebsiella pneumoniae*, *Acinetobacter baumannii*, *Pseudomonas aeruginosa*, and *Enterobacter* spp., which are collectively referred to as ESKAPE [1]. ESKAPE pathogens have shown varying levels of resistance to antibiotics [2,3,4]. In a Peruvian hospital, the use of ceftazidime was correlated with an increase in carbapenem-resistant *Pseudomonas aeruginosa* isolations and resistance to piperacillin/tazobactam in *Enterobacter* spp. and ciprofloxacin usage [1]. In a Romanian infectious diseases hospital, high rates of multi-drug resistance were observed in methicillin-resistant *Staphylococcus aureus*, extended-spectrum beta-lactamase-producing *Enterobacterales*, carbapenem-resistant *Pseudomonas aeruginosa*, *Acinetobacter baumannii*, and *Klebsiella pneumoniae* [5]. A study in Nepal found a high percentage of drug resistance and biofilm production among ESKAPE pathogens, highlighting the need for antimicrobial resistance surveillance [6].

Bacterial organisms are scarcely observed residing in a planktonic state; instead, they tend to safeguard their communities from deleterious elements via the generation of spatial patterns enveloped by an extracellular matrix known as biofilm [5]. Biofilm is a complex, stratified structure comprising organized microorganisms that attach to moist surfaces. The bacterial cells within this structure exhibit a high degree of coordination, leading to the formation of a community that is capable of withstanding harsh external conditions. This resilience is further bolstered by genetic mutations and gene transfers that promote resistance to antibiotics [7]. *A. baumannii*, denoted by the letter “A” in the acronym “ESKAPE”, is a Gram-negative opportunistic and nosocomial pathogen. It has the potential to produce biofilm and cause infections in both individuals who have been hospitalized for extended durations as well as those who have been exposed to prolonged antimicrobial therapy [8,9,10]. It has been observed that *A. baumannii*, which is resistant to antibiotics, exhibits a robust ability to form biofilms [11]. The ability of bacterial cells to coordinate their gene expression through quorum sensing (QS) allows for the development of a multi-drug resistance feature that enables adaptation to change in environmental conditions, specifically in the form of biofilm [12]. A biofilm that has reached full maturity and development poses a considerable challenge when it comes to eradication, requiring specific compounds capable of penetrating its complex structure or mechanically disrupting it [13]. The functional efficacy of conventional drugs is diminished by the swift emergence of drug resistance in microorganisms. This resistance has prompted researchers to explore the potential of nanoparticles as components of an alternative strategy in addressing the challenge of highly aggressive pathogens such as *Acinetobacter*. Furthermore, no evidence of inherent resistance in bacteria towards organic and inorganic nanoparticles has been detected, which sets them apart from conventional antibiotics [14].

Nanoparticles (NPs), which are highly prevalent in natural surroundings and possess distinct characteristics, have demonstrated an ability to combat bacterial infections through various mechanisms such as direct interaction with the bacterial cell wall, the inhibition of biofilm formation, the activation of innate and acquired host immune responses, the generation of reactive oxygen species (ROS), and interaction with DNA and/or proteins [15]. Using nanoparticles (NPs) has been demonstrated in various studies to be a highly promising technique for addressing the challenge of microbial drug resistance [16]. The physical and chemical attributes of nanostructures, including their size, surface charge, and solubility, whether organic or inorganic, have been observed to exhibit antimicrobial properties [17,18]. Mesoporous silica nanoparticles (MSNs) possess various properties that make them ideal therapeutic nanocarriers. Their large surface areas and pore volumes make them suitable for drug loading. Additionally, their adjustable morphologies, excellent biocompatibility, and capacity to release pharmaceuticals in response to external stimuli are advantageous. MSNs also have the potential for medicinal synthesis in conjunction with other medications due to their functionalization advantage. By targeting specific sites for drug transactions, their adverse effects can be significantly lowered, and more extensive drug loading can directly increase therapeutic efficacy. Furthermore, MSNs offer other notable advantages such as simple and inexpensive synthesis and stability [19]. Due to their capacity to confer solubility and stability to pharmaceuticals in solutions, MSNs have garnered attention in the scientific community.

Silica nanoparticles have shown promise in inhibiting biofilm formation. Hetrick et al. demonstrated that nitric oxide (NO)-releasing silica nanoparticles were able to kill biofilm-based microbial cells, including *Pseudomonas aeruginosa* and *Escherichia coli* biofilms, with a killing efficacy of ≥99.999% [20]. Türetgen also investigated using nano-silica coatings on cooling tower fill materials and heat exchangers to reduce biofilm formation. In both studies, the nano-silica coatings significantly reduced biofilm formation, with up to a 4 log reduction in surface-associated bacteria on coated samples compared to uncoated controls [21,22,23]. Additionally, Devlin et al. found that *Pseudomonas putida* biofilms showed enhanced entrapment efficiencies for silica nanoparticles compared to *Pseudomonas fluorescens* biofilms regardless of nanoparticle size or surface functionalization [24]. These findings suggest that silica nanoparticles have the potential to be effective against *Actinobacter* biofilm formation.

In recent times, *A. baumannii* has emerged as the topmost concern owing to its (MDR) nature [25]. This bacterium exhibits diverse metabolic states [26], especially when it grows as biofilm, which renders it capable of withstanding a wide range of chemical agents. Thus, identifying novel molecules that can combat this pathogen has become a matter of utmost significance. In this regard, our research provided pioneering therapeutic approaches in the form of different silica nanoparticle treatments that have been made for the first time to address *A. baumannii* biofilm. Therefore, the present study aimed to investigate the impact of these novel nanoparticles on the biofilm produced by *A. baumannii* and to compare these different NP modules in terms of their efficacy in biofilm inhibition. Moreover, to the best of our knowledge, this is the first study to hybridize amoxicillin and silica nanoparticles to inhibit *Acinetobacter* biofilm formation. Our research findings could potentially lead to the development of innovative treatments to combat *Acinetobacter* infections and reduce the morbidity and mortality associated with MDR bacterial infections.

## 2. Material and Methods

### 2.1. Sample Preparation

The Acinetobacter baumannii American Type Culture Collection (ATCC 19606) strain used in this study was acquired from Dr. Ola Al-Sanabra, a member of the Faculty of Science at Al-Balqa Applied University. For bacterial growth, aerobic inoculation was carried out in BHI broth supplemented with 2% glucose and 2% sucrose at 37 °C for 24 h.

### 2.2. Amoxicillin Preparation

*A. baumannii* strains exhibit high resistance to amoxicillin in vivo, primarily attributed to their ability to form biofilms [27]. The growth of bacterial strains in these biofilms needs a dose that is 250 times the antibiotic concentration required to prevent the growth of the same strains when grown planktonically in in vitro studies [28]. In this study, amoxicillin was employed in two ways. Firstly, it was used as a positive control with a concentration more than twice that recorded in the experiment by [28] to ensure complete biofilm inhibition. Secondly, it was used in small doses in combination with silica nanoparticles to demonstrate the capability of our novel nanoparticles in addressing the resistance caused by biofilm formation. This approach can pave the way for developing effective strategies to combat antibiotic resistance in bacterial strains.

To prepare the stock solution, a quantity of Amoxicillin weighing 0.1 g was dissolved in 10 mL of distilled water, to which 2 drops of ethanol were added. The solution was then placed on a shaker at a temperature of 37 °C while being agitated at a rate of 200 rpm for 30 min. This stock solution was subsequently used to prepare the various concentrations required for the individual experiments in this study.

### 2.3. Nanoparticle Preparation

Diatomaceous earth, which is composed of SiO_2_ and celite 545, was procured from Sigma-Aldrich in Germany and used as the base for the production of the other treated silica nanoparticles used in this research.

The initial treatment of silica nanoparticles involved the preparation of CuO.SiO_2_ composite via the amalgamation of 25.0 g of micro-sized copper oxide (CuO) and 25.0 g of SiO_2_ in a 6.0 M (NaOH) solution of 100.0 mL according to the procedure outlined in [29]. The resultant paste was subjected to a temperature of 70 °C for 24 h, followed by filtration and washing with deionized water, distilled water, and HCl. The CuO.SiO_2_ particles were subsequently dried in an oven at 60 °C.

To create alkali silica NaOH.SiO_2_ nanoparticles, a sample of 5.5 g of diatomite was boiled with a 20 mL solution containing 3.8 g NaOH for 2 h. The solid was filtered, washed with distilled water, and dried at 80 °C in the oven.

Finally, H_3_PO_4_.SiO_2_ was prepared by blending 33.0 g of diatomite sample with 50 mL of 8.0 M NaOH in a plastic container, sealing the mixture, and placing it in an oven at 70 °C for 24 h. The resulting mixture was then combined with 150 mL of water, decanted to obtain a clear solution, and neutralized with H_3_PO_4_ to attain a pH of 7. The mixture was left to form a gel, which was entirely transparent. Subsequent washing with acetone resulted in a dry gel that was subsequently powdered.

Upon the completion of preparatory measures, it was necessary to evaluate the pH value of all treatments to ensure that the growth of bacteria was not impeded due to alterations in the medium’s acidity, as bacteria prefer to live in a neutral medium. To guarantee the accuracy of this assessment, equal volumes of BHI broth media were supplemented with 2% glucose and 2% sucrose, each nanoparticle treatment was mixed, and the final outcomes were documented using a pH meter. Following this, all previously mentioned treatments were utilized in the procedures for biofilm inhibition. It is essential to ensure that the pH value of the medium is maintained at a neutral level to support bacterial growth.

#### 2.3.1. Nanoparticle Characterization

##### Scanning Electron Microscope

Scanning electron microscopy (SEM) was used to investigate the morphology of silica nanoparticles.

##### Silica Nanoparticles’ Cytotoxicity against Normal Cell Line

In order to guarantee the safety characteristics of silica nanoparticles, our research team analyzed the potential cytotoxic consequences of silica nanoparticles for normal human cells, which involved the assessment of the viability values of human fibroblast cell line when incubated with the different silica nanoparticles.

Following [30]’s procedure, the 3-(4,5-dimethylthiazol-2-yl)-2,5-diphenyltetrazolium bromide (MTT) dye reduction test was implemented to determine the growth of normal cells. Normal fibroblast cells (50 μL, 1 × 10^5^ cells/mL) were seeded on 96-well plates. These cells were then subjected to varying concentrations (from 2 mg/mL to 0.06 μg/mL) of serially diluted SiO_2_, CuO.SiO_2_, H_3_PO_4_.SiO_2_, and NaOH.SiO_2_ and incubated for 24 h at 37 °C and 5% CO_2_. Following this, 10 μL of MTT (0.5 mg/mL) was introduced to each well and incubated for a duration of 4 h. After this incubation, the cells were lysed using 10% sodium dodecyl sulfate (SDS) in 0.01 M hydrochloric acid (HCl) solution. The absorbance of each well was measured at 570 nm using an enzyme-linked immunosorbent assay (ELISA) reader for microplates and the data were recorded.

### 2.4. Determination of Minimal Biofilm Inhibitory Concentrations (MBICs) by Using Microtiter Plate Method

#### 2.4.1. Microtiter Plate Biofilm Formation Assay

The microtiter plate biofilm formation assay is a laboratory technique used for measuring biofilm formation by microorganisms that involves using multi-well plate and staining methods to assess the adherence and growth of microbial cells on a surface [31].

To create biofilms, which are often viewed as a negative control, a specialized microtiter plate made from sterile, flat-bottomed polystyrene containing 96 wells with a lid was implemented. Following the procedure described by [32], 200 μL of the bacterial broth, equivalent to 0.5 McFarland turbidity, was transferred to each well of the microtiter plate. After the incubation period of 24 h, the suspensions were removed, and the non-adherent bacteria were washed with sterile distilled water several times. The wells were then dried for 45 min. Subsequently, crystal violet stain (0.5% *w*/*v*) was added to each well for 45 min. To remove excess stains, the wells were washed five times with sterile distilled water. Finally, 95% ethanol was added to each well to eliminate the dye that emerged from adherent cells. The bacterial biofilm was evaluated by scanning the plate using an ELISA reader at 570 nm. The experiment was conducted in triplicate.

##### Evaluation of Different Media for Biofilm Formation

In this particular investigation, the previous microtiter plate method was utilized to evaluate three distinct categories of media to detect biofilm formation by A. baumannii. These media are BHI medium, LB medium, and DMEM/N medium; all these media were supplemented with 2% glucose and 2% sucrose, and LB media without any additional supplements constituted the third category. The bacteria were grown in the media mentioned above for 20 to 24 h at a temperature of 37 °C. The results were analyzed after incubation lasting 20 to 24 h, followed by plate washing several times with sterile distilled water and staining using crystal violet stain as mentioned in the biofilm formation assay. The growth of bacteria was assessed by utilizing an enzyme-linked immunosorbent assay (ELISA) reader at 570 nm.

#### 2.4.2. Microdilution Assay

In order to assess the effectiveness of different treatments in preventing the formation of biofilms, a microdilution assay was carried out using established experimental protocols with minor modifications as outlined by [33,34]. For each well of the plate, 200 μL of bacterial broth was added. These broth wells were then treated with varying concentrations (ranging from 1 mg/mL to 0.06 μg/mL) of serially diluted SiO_2_, CuO.SiO_2_, H_3_PO_4_.SiO_2_, and NaOH.SiO_2_. A combination of nanoparticles and amoxicillin, in a ratio of 1:1, was employed in this assay. This means that for every 1 mg/mL of pure silica, 1 mg/mL of amoxicillin was combined, and so on for all the treatments. In addition, prior to adding the combined treatments to the broth, they underwent a twofold dilution ranging from a ratio of 1:1 mg/mL to a ratio of 0.06:0.06 μg/mL. Then, all treatments (both combined and not combined) were subsequently incubated for 24 h at 37 °C. The negative control involved no treatments, resulting in equal biofilm formation in each well. In contrast, the positive control was treated with amoxicillin alone, which underwent a series of 2-fold dilutions from 5 mg/mL to 0.06 μg/mL and then was processed similarly to the other treatments in this assay. After these steps, plates were washed and stained with crystal violet, as in the previous biofilm formation experiment. Finally, the density of the adherent bacteria was measured by determining the absorbance of each well at 570 nm using a microtiter ELISA reader. The lowest concentration of silica nanoparticle treatment necessary to inhibit bacterial biofilm formation was determined as the minimal biofilm inhibitory concentration (MBIC). We conducted a preliminary experiment to evaluate the effectiveness of these innovative nanoparticles and, thus, did not establish the minimum biofilm eradication concentration (MBEC). Instead, we compared the absorbance values for each MBIC to ensure that they were significantly equivalent in inhibition despite differences in their MBIC values. With the assistance of a spectrophotometer, we measured the biofilm density of MBIC value for each silica treatment against that of the negative and positive control absorbances. This process was repeated three times to ensure the accuracy and reliability of the outcomes.

#### 2.4.3. Confirmation Step

The efficacy of silica treatments in inhibiting biofilm formation was confirmed by using an inverted microscope for screening biofilm inhibition. To conduct the experiment, a bacterial suspension with a McFarland turbidity of 0.5 was taken in six sterile flask tubes, each containing 2500 μL. One of the tubes was designated as a negative control without any treatment, and one, as a positive control, was treated with 5 mg/mL of amoxicillin. The other tubes were treated with 1 mg/mL from each silica nanoparticle treatment (pure, composite, alkali, or H_3_PO_4_ silica). All the flasks were incubated at 37 °C for 20–24 h with a loose flask lid. Subsequently, the flasks were washed and stained with crystal violet, like in the previous biofilm formation experiment, with attention paid to differences in reagent volumes. The adherent bacterial biofilm within the flasks was finally visualized using an inverted microscope.

### 2.5. Statistical Analysis

The results were described in terms of means and standard error (SEM). For the statistical analysis, a one-way analysis of variance was performed (ANOVA). Calculations were performed using GraphPad Software (Boston, MA, USA). Each experiment was performed in triplicates.

## 3. Results

### 3.1. Nanoparticle Characterization

#### 3.1.1. Nanoparticle Morphology

**Figure 1:**
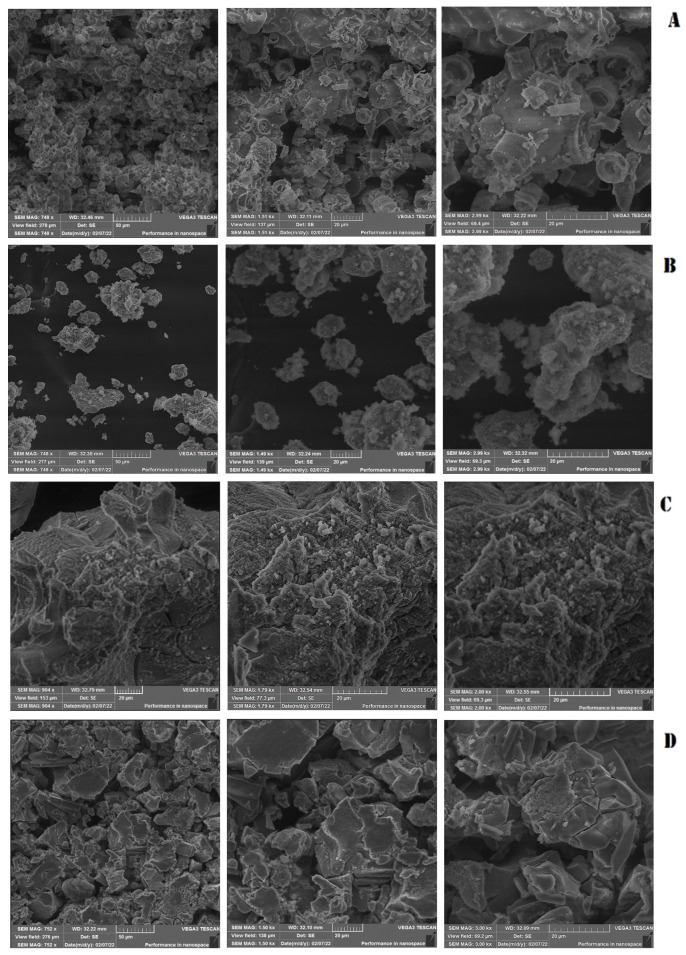
Scanning electron microscopy (SEM) of silica nanoparticles. (**A**) Pure silica without treatment. (**B**) Silica nanoparticles treated with CuO. (**C**) Silica nanoparticles treated with NaOH. (**D**) Silica nanoparticles treated with H_3_PO_4_. Moving from left to right, the magnification progressively increases.

The image presented in Figure 1A shows pure silica nanoparticles, also known as diatomaceous earth, which possess a distinctive cylindrical structure. This structure exhibits a length of approximately 15 to 20 μm while the internal pore diameter of these nanoparticles is estimated to be around 4 μm. The morphology of these nanoparticles is a critical factor that plays a significant role in their properties and behavior.

The amorphous irregular shapes of CuO.SiO_2_ and NaOH.SiO_2_ nanoparticles, as shown in Figure 1B,C, affect their surface areas, reactivity, and other physicochemical characteristics, making them potentially suitable for various applications in fields such as catalysis and biomedicine. The morphology of the H_3_PO_4_.SiO_2_ nanoparticles, as illustrated in Figure 1D, show a configuration that takes the form of silica sheets. The morphology of nanoparticles, including factors such as their shape, size, and surface area, significantly impacts their physical and chemical behavior, making them ideal for various applications. In this regard, our research team utilized nanoparticle modulation to effectively impede biofilm formation while simultaneously functioning in synergy with Amoxicillin medication to effectively combat the aforementioned biofilm.

#### 3.1.2. Medium Acidty Confirmation Assay

Before the synthesis of nanoparticles, the reagent NaOH was employed, while NaOH and H_3_PO_4_ were utilized as treatments for the nanoparticles. To ensure the successful neutralization of these particles post-synthesis, a pH test was conducted on all treated nanoparticles. The results of the study clearly indicated that the effects on the pH levels by all the nanoparticles used, including NaOH, CuO, H_3_PO_4_, and pure silica, were within the neutral range, with values of 7.30, 6.70, 6.61, and 6.50, respectively. This successful implementation of neutralization is a crucial step towards producing high-quality NPs for various applications, such as in our study.

#### 3.1.3. Silica Nanoparticles Cytotoxicity against Normal Cells

In the current investigation, our findings indicate that silica nanoparticles, when treated with CuO, NaOH, and H_3_PO_4_ separately, can target *Actinobacter* biofilms without causing harm to normal cells. To evaluate the possible cytotoxicity of these nanoparticles, several concentrations ranging from 2 mg/mL to 0.06 μg/mL were tested.

Our results demonstrated that the concentration of 2 mg/mL of silica treated with NaOH was the maximum safe concentration of all nanoparticles, recording 73% viability compared to other concentrations of the same treatment and other treatments. The concentration of 1 mg/mL of pure silica, which registered 71% viability, followed NaOH treatment.

**Figure 2:**
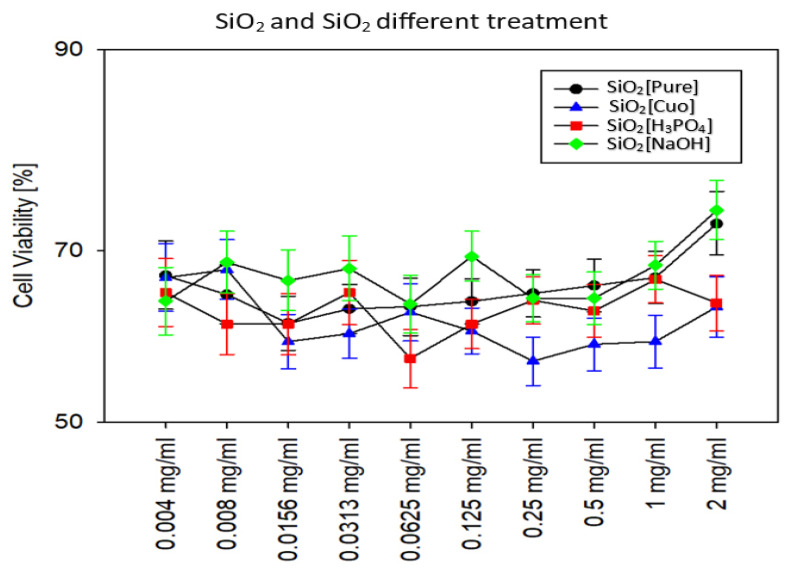
Viability test of normal fibroblast cell line after different silica NP treatments.

Although H_3_PO_4_- and CuO-treated nanoparticles reported the lowest viability compared to the rest of the nanoparticles in this study, they are still relatively safe since they have a viability percentage over 50%. The highest viability value of H_3_PO_4_ was at the concentration of 1 mg/mL, while the highest viability value for CuO treatment that registered the lowest safety nanoparticles in this study was at the concentration of 0.008 mg/mL, as shown in Figure 2. For this reason, 1 mg/mL was the highest concentration chosen to be combined with Amoxicillin in the further experiments in this study.

### 3.2. Evaluation of Media for Biofilm Formation In Vitro

**Figure 3:**
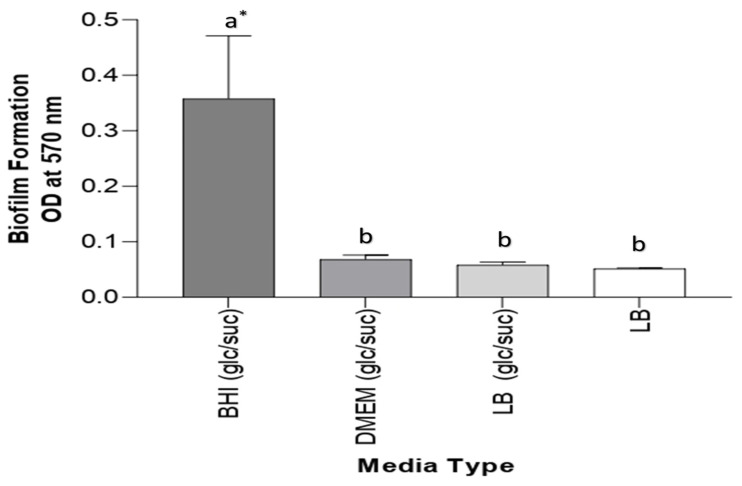
Biofilm formation of A. *baumannii* in different types of media. The result represents that BHI (glc/suc) media have the highest biofilm formation compared to other media. * Values represent means ± standard error. Statistical analysis involving different types of media was performed using a one-way ANOVA followed by a Tukey multiple comparison post-test, * *p* <0.05. Different letters indicate significant comparison. glu/suc: glucose/sucrose.

Three different media were examined for biofilm formation: DMEM and BHI supplemented with 2% glucose and 2% sucrose and LB media with 2% glucose and 2% sucrose and without supplementation. The effectiveness of all used media was examined. BHI medium containing 2% glucose and 2% sucrose exhibited considerably strong biofilm formation; its absorbance was equal to 0.350. However, weak biofilm formations using DMEM and LB-supplemented media had absorbance values of 0.068 and 0.058, respectively. Non-supplemented LB media obtained the lowest absorbance value, 0.051, for biofilm formation; because of this result, BHI medium was employed for the other experiments. All data are described in Figure 3.

### 3.3. The Effect of Different Treatments on Biofilm by Scanning Optical Density Using Microtiter Plate

**Figure 4:**
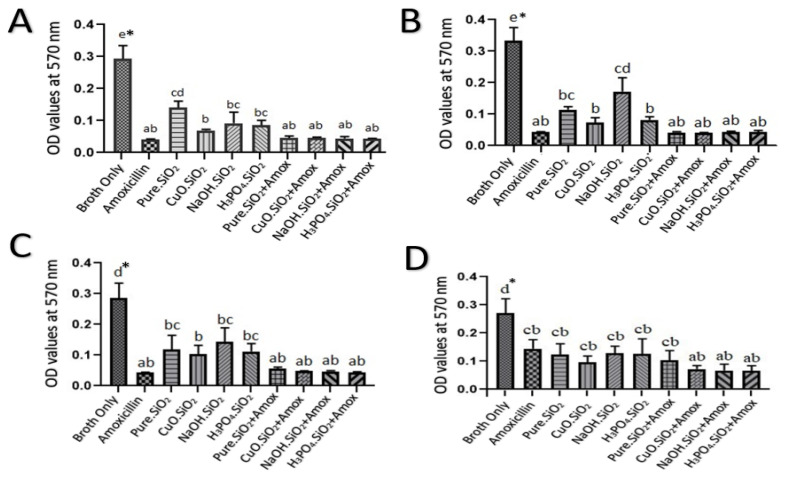
The effects of various silica NP treatments on *A. baumanni’s* biofilm inhibition at concentrations of (**A**) 1.0 mg/mL, (**B**) 0.5 mg/mL, (**C**) 0.25 mg/mL, and (**D**) 0.125 mg/mL. The negative control represents broth only, and the positive control represents (**A**) 5.0 mg/mL amoxicillin, (**B**) 2.5 mg/mL amoxicillin, (**C**) 1.25 mg/mL amoxicillin, and (**D**) 0.625 mg/mL amoxicillin. * Values represent means ± standard error. Statistical analysis involving different treatments was performed using a one-way ANOVA followed by a Tukey multiple comparison post-test, * *p* < 0.05. Different letters indicate significant comparison.

The results of the bacterial biofilms that were treated with a 5.0 mg/mL amoxicillin as a positive control and a 1.0 mg/mL from each of the four silica treatments separately demonstrated that the absorbance values were 0.04, 0.13, 0.07, 0.09, and 0.08, for pure silica, CuO.SiO_2_ composite, alkali silica, and H_3_PO_4_.SiO_2_, respectively. These values are significantly lower than the absorbance value for the negative control (0.3) (*p* < 0.05). On the other hand, the inhibition had increased according to the absorbance values from 0.3 to 0.04 when amoxicillin was combined with each silica NP treatment, as shown in Figure 4A.

The results that illustrated, in Figure 4B, absorbance values for each silica treatment at a concentration of 0.5 mg/mL showed that bacterial biofilm, when compared with 2.5 mg/mL amoxicillin, was inhibited to 0.04, 0.11, 0.07, 0.16, and 0.08, for pure silica, CuO.SiO_2_ composite, alkali silica, and H_3_PO_4_.SiO_2_, respectively. Interestingly, the combination of these NPs with amoxicillin showed an effective reduction in the absorbance values from 0.3 to 0.042.

The inhibition effect on bacterial biofilm, which was indicated by the absorbance in response to each treatment of 1.25 mg/mL amoxicillin and other NPs at a concentration of 0.25 mg/mL, showed that the biofilms were reduced by the combination of amoxicillin and NPs from 0.3 to 0.043 while the absorbance of pure silica was 0.12 and the absorbance values of CuO.SiO_2_ composite and H_3_PO_4_.SiO_2_ were 0.10 and 0.11, respectively. However, alkali silica produces the highest absorbance value (0.14), which indicates the lowest inhibition efficacy, and this is revealed in Figure 4C.

Figure 4D represents that at a concentration of 0.125 mg/mL, all silica treatments still have antibacterial effects to inhibit the biofilm. The data showed that 0.625 mg/mL amoxicillin has the highest absorbance value compared to other treatments, which have inhibition values, ranging approximately from 0.3 to 0.13, that are induced by pure silica, alkali, and H_3_PO_4_.SiO_2_ NPs. Meanwhile, composite NPs reduced the absorbance values from 0.3 to 0.09. In addition, the mixture of amoxicillin and silica NPs at this concentration revealed the highest inhibition—from 0.3 to 0.07—for composite, alkali, and H_3_PO_4_.SiO_2_ mixtures, while the absorbance value of the combination between pure silica and amoxicillin was 0.10.

### 3.4. The Effects of Different Treatments on Biofilm Using Cell Culture Flask Tubes

**Figure 5:**
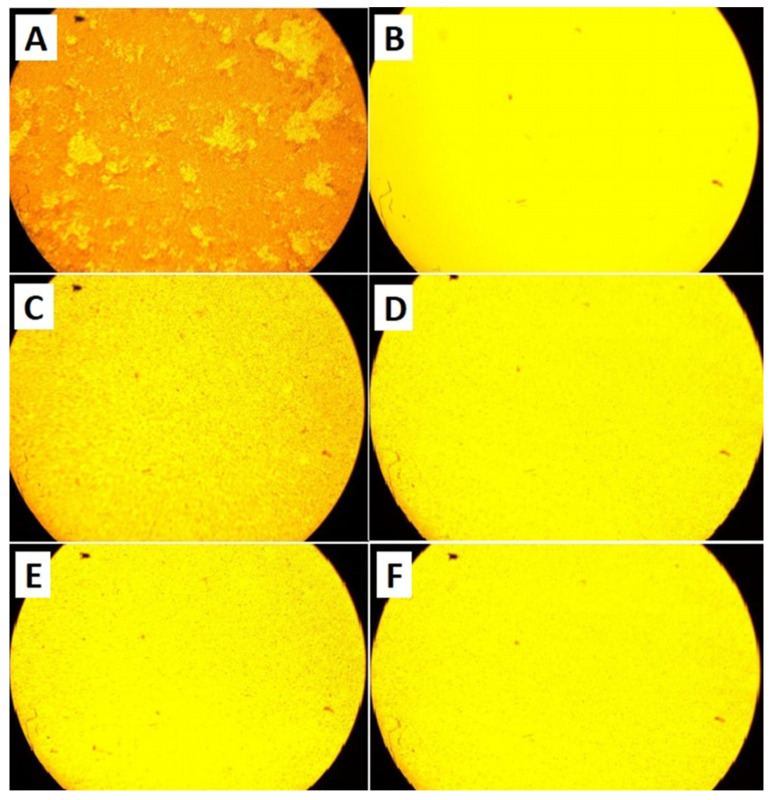
Microscopic image under 10× magnification power. (**A**) *A*. *baumannii* biofilm without treatment. The bacterial biofilm formed in a flask tube after 20–24 h at 37 °C. (**B**) Amoxicillin’s effect on bacterial biofilm. The antibiotic amoxicillin caused the complete inhibition of bacterial biofilm following treatment with a concentration of 5.0 mg/mL for 20–24 h and incubation at 37 °C. (**C**) *A*. *baumannii* biofilm treated with pure silica NPs. The bacterial biofilm was inhibited following treatment with the 1.0 mg/mL of pure silica NPs for 20–24 h at 37 °C. (**D**) *A. baumannii* biofilm treated with composite CuO.SiO_2_ NPs. The bacterial biofilm was inhibited following treatment with 1.0 mg/mL of composite CuO.SiO_2_ NPs for 20–24 h at 37 °C. (**E**) *A. baumannii* biofilm treated with alkali silica NPs. The bacterial biofilm was inhibited following treatment with the 1.0 mg/mL of alkali NPs for 20–24 h at 37 °C. (**F**) *A. baumannii* biofilm treated with H_3_PO_4_.SiO_2_ NPs. The bacterial biofilm was inhibited following treatment with 1.0 mg/mL of H_3_PO_4_.SiO_2_ NPs for 20–24 h at 37 °C.

As previously mentioned, the negative control consisted solely of bacterial broth with no treatments, serving to indicate in vitro biofilm formation. The bacterial biofilm was examined under an inverted microscope, as depicted in Figure 5A, revealing complete biofilm formation. The evaluation of bacterial biofilm inhibition utilizing the positive control, amoxicillin, at a concentration of 5.0 mg/mL resulted in the complete inhibition of the bacterial biofilm, as demonstrated in Figure 5B; upon the application of 1.0 mg/mL of pure silica NPs to bacterial biofilm, biofilm inhibition was observed, as presented in Figure 5C; furthermore, the treatment of bacterial biofilm with 1.0 mg/mL of composite CuO.SiO_2_ also resulted in biofilm inhibition, as illustrated in Figure 5D. Finally, H_3_PO_4_ and alkali silica treatment at a 1.0 mg/mL concentration exhibited biofilm inhibition, as depicted in Figure 5E and Figure 5F, respectively.

### 3.5. Determination of Minimum Biofilm Inhibition Concentration (MBIC)

**Figure 6:**
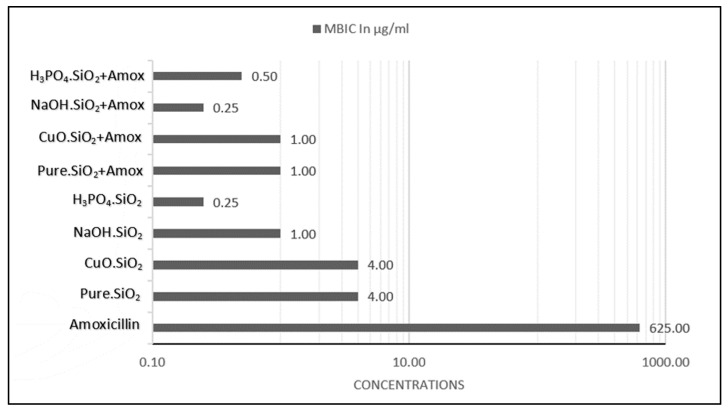
MBIC values in µg/mL for amoxicillin and other NP treatments. Amoxicillin has the highest MBIC value compared to other silica NP treatments.

**Figure 7:**
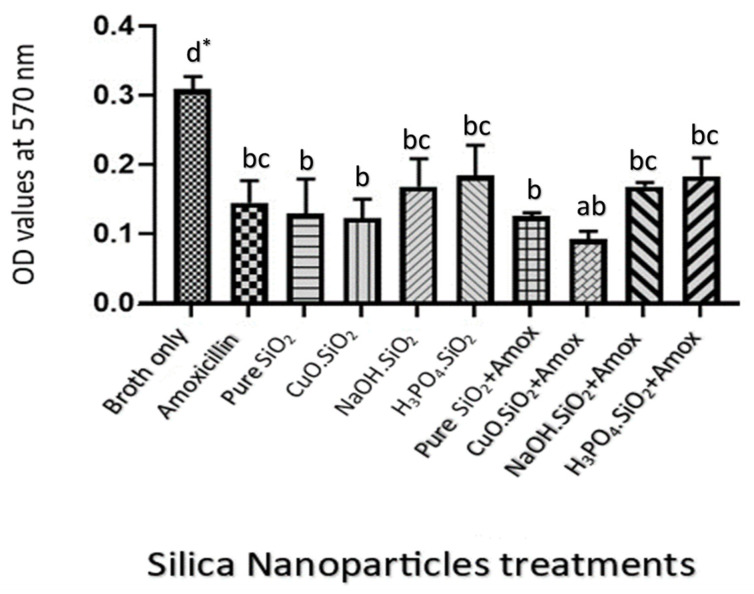
Differences between MBIC values for different NP treatments and positive control. There were no significant differences in inhibition according to absorbance values. Negative control represents broth only; positive control represents amoxicillin. * Values represent means ± standard error. Statistical analysis involving different treatments was performed using a one-way ANOVA followed by a Tukey multiple comparison post-test, * *p* < 0.05. Different letters indicate significant comparison.

The minimum biofilm inhibition concentration (MBIC) of amoxicillin alone was 625 µg/mL, which is the minimum concentration that inhibited the reform of bacterial biofilm, as shown in Figure 6. The MBIC values that caused the inhibition of bacterial biofilm by pure silica NP treatment, composite CuO.SiO_2_, alkali silica treatment, and H_3_PO_4_.SiO_2_ NPs were 4.0, 4.0, 1.0, and 0.25 µg/mL, respectively. The MBIC values for the combination of pure silica NPs and amoxicillin, composite NPs and amoxicillin, alkali NPs and amoxicillin, and H_3_PO_4_.SiO_2_ NPs with amoxicillin were 1.0, 1.0, 0.25, and 0.50 µg/mL, respectively. It is possible that the absorbance levels for various MBIC values may vary based on their inhibiting efficacies. In case the MBIC for pure silica is lower than that of a positive control, it is likely that the positive control may have a larger inhibition area in the MBIC compared to that of pure silica. To ensure that the differences in MBIC had significantly similar biofilm inhibition efficacies, we measured the differences in absorbance values. The results demonstrated no significant changes (*p* < 0.05) in the effectiveness of the treatment concentrations in inhibiting bacterial biofilm despite the differences between the MBIC values of each treatment, as illustrated in Figure 7.

## 4. Discussion

The aim of this study was to establish and control bacterial biofilm formation in vitro. Certain bacteria have the ability to form biofilms for persistence and survival. Our results showed that the BHI medium, with an absorbance value of 0.35, was better than the other tested media (LB and DMEM/N) for biofilm production, which had values of 0.058 and 0.068 (respectively), as shown in Figure 3. BHI medium has been utilized in numerous biofilm formation studies. While BHI medium supplemented with 10% human plasma has exhibited reproducible and robust *Staphylococcus aureus* biofilm, it has displayed unfavorable outcomes for the biofilm formation of *Pseudomonas aeruginosa*, *Acinetobacter baumannii*, and *Klebsiella pneumoniae* in vitro. The results of [35] did not correlate with our own, as BHI was the most effective medium for *Acinetobacter baumannii* biofilm formation. However, *S. haemolyticus* biofilm production can be increased by cells grown in BHI broth containing glucose, and this result aligns with our assumption that 2% glucose supplementation may enhance biofilm formation in vitro [36]. Therefore, it is imperative to acknowledge that the utilization of a BHI medium for biofilm formation may fluctuate depending on the specific experimental conditions and bacterial strain being examined [37]. Overall, BHI medium has demonstrated efficacy for biofilm formation in various studies, potentially due to the sugar supplement and high nutrient composition of BHI medium compared to other basic media, such as dextrose, which serves as an energy source. Additionally, BHI medium contains protease peptone and infusions (calf brain and beef heart) necessary for nitrogen compounds, carbon and growth factors, amino acids, and vitamins. In contrast, other types of basic media possess fewer nutrients essential for bacterial growth, and these findings coincide with those of [38,39], who recommended BHI medium as an excellent culture medium for biofilm formation.

Moreover, this study was conducted to evaluate the effects of stable, biocompatible, and less-toxic novel green silica NPs on bacterial biofilm and to figure out whether there were differences between these silica NP treatments in relation to biofilm inhibition efficacy.

The utilization of scanning electron microscopy has enabled the examination of the cylindrical structure of pure silica nanoparticles, as illustrated in Figure 1. This structure is characterized by a length ranging from approximately 15 to 20 μm, with an internal pore diameter of 4 μm. The morphology of mesoporous nanoparticles is a crucial factor that significantly influences their properties and behavior. Specifically, mesoporous silica nanoparticles (MSNs) have been employed in several studies to inhibit biofilm formation. For instance, in one study, MSNs encapsulated with benzalkonium chloride (BAC) exhibited remarkable efficacy in inhibiting the growth of *Listeria monocytogenes*, a targeted bacterium, even at lower disinfectant concentrations. Interestingly, our results align with these findings, showing that small doses of antibiotics were more effective in inhibiting biofilm formation when combined with silica nanoparticles as opposed to when they were used alone [40]. In another study, the focus was on using thin-film coatings embedded with gentamicin-loaded MSNs (MSN-G) to achieve prolonged antibacterial and anti-biofilm activity. The MSN-G films were characterized by controlled and slow gentamicin release, which resulted in prolonged antibacterial efficacy against *Staphylococcus aureus* and the persistent prevention of bacterial growth for more than two months [41]. Additionally, SiO_2_ mesoporous nanosystems loaded with essential oils, including eucalyptus, orange, and cinnamon, were developed and tested for their antibacterial and anti-adherence effects. Notably, these nanosystems exhibited the potential to inhibit the growth of *Staphylococcus aureus*, *Escherichia coli*, and *Candida albicans* [42]. Overall, these studies underscore the potential of MSNs in inhibiting biofilm formation and controlling bacterial growth.

The impact of NaOH used in manufacturing on the porous structure of pure silica was observed in that it dissolved silica, leading to the precipitation of amorphous-shaped material in NaOH- and CuO-treated silica nanoparticles. This effect caused the destruction of the porous structure, observed using SEM, of pure silica (Figure 1B,C).

Metal nanoparticles have been extensively studied for their potential applications in various fields including biology and therapeutics. Using metal oxide nanoparticles (NPs) has shown significant anti-biofilm activity, as demonstrated in the studies by [43,44,45,46,47]. These NPs possess antimicrobial properties, which can inhibit the formation of biofilms. Among the metal oxide NPs, Zinc Oxide (ZnO) NPs have been found to exhibit antibacterial activity against a variety of bacteria including *Streptococcus pyogenes*, *Bacillus cereus*, *Escherichia coli*, and *Pseudomonas aeruginosa*. These NPs disrupt the cell membranes of bacteria, leading to the leakage of nucleic acid and subsequent inhibition of biofilm formation. Thus, using metal oxide nanoparticles can potentially be a promising approach to prevent biofilm-related infections. Moreover, metal oxide nanoparticles (NPs) have shown potential in inhibiting quorum sensing, a process involved in biofilm formation in bacteria like *Pseudomonas aeruginosa*. This discovery has made metal oxide NPs promising candidates for combating biofilm formation, and they can be used as effective anti-biofilm agents. The safety of metal nanoparticles, however, remains a crucial concern, as they must be biocompatible and exhibit low or no toxicity when interacting with the human body [48]. The toxicity of metal nanoparticles can be influenced by various factors such as their physicochemical properties, behavior, and biological toxicity. It is stated in [49] that metal oxide nanoparticles have been extensively studied for their toxicity and potential adverse effects on living organisms and cells. These nanoparticles can enter the body through different exposure routes including injection, inhalation, skin penetration, and ingestion [50]. In addition, metal oxide nanoparticles are a subject of increasing concern due to their potential toxicity. Several factors, such as particle size, surface charge, and concentration, can influence their toxicity [51]. Exposure to these nanoparticles can result in various harmful effects on endothelial cells, including phenotypic changes, oxidative stress, and apoptosis [52]. The production of reactive oxygen species and the dysfunction of nitric oxide synthase are also linked to the toxicity of metal oxide nanoparticles [53]. Quantum-mechanical descriptors, such as the enthalpy of formation and Fermi energy, can be utilized to elucidate the toxicity of these nanoparticles [54]. Overall, metal oxide nanoparticles can exert their toxicity through different mechanisms, and this correlated with our results in that CuO-treated nanoparticles recorded the highest cell toxicity against normal fibroblast, as shown in Figure 2. In our investigation, we examined silica nanoparticles subjected to copper oxide treatment on a micro scale. Our primary concern was to ascertain both the safety profile of these novel nanoparticles post-treatment as well as the safety profiles of other treatments used in this study. As a result, we decided to conduct a cytotoxicity analysis of these nanoparticles against normal fibroblast cell lines to ensure their safety profile. Our findings have demonstrated the safety profile of these nanoparticles, as the viability of fibroblast cells ranged from 61% to 73% on average following treatment with these nanoparticles. In our study, we also observed that the composite CuO.SiO_2_ and its combination with the antibiotic exhibited significant inhibition of bacterial biofilm. This could be due to various mechanisms, such as those observed in other metal NPs, including AgNPs and AuNPs, which release numerous biomaterials and metal ions during such an interaction, thereby enhancing antibacterial activity and mechanical behavior. Hence, our study has provided valuable insights into the cytotoxicity and antibacterial properties of these novel nanoparticles [16,55].

When alkaline substances such as sodium hydroxide (NaOH) are used to modify silica nanoparticles (NPs), a change in pH leads to the production of more purified amorphous NPs. However, this change in pH during manufacturing does not affect the pH of the bacterial medium after post-treatment with either NaOH.SiO_2_ or H_3_PO_4_.SiO_2_ of bacterial biofilm. This was confirmed by measuring the pH of the BHI medium after mixing these NPs with it. As a result, the efficacy of these NPs, not the pH change, led to the inhibition of bacterial biofilm. This finding is consistent with the results of Luthfiah [56], which revealed that more alkaline silica is effective in obtaining material of high purity. Thus, it can be concluded that using alkaline-modified silica NPs can be an effective means of inhibiting bacterial biofilm without altering the pH of the bacterial medium. Further research is warranted to investigate the full potential of these NPs in medical and industrial applications.

Treating bacteria with silica nanoparticles (NPs) modified by phosphoric acid H_3_PO_4_ can increase the concentration of hydrogen ions present, resulting in a loss of vitality and reduced biofilm formation. These findings are consistent with those reported by Prado and co-workers [57]. It is evident that the use of modified NPs can significantly impact the viability and biofilm formation of bacteria, highlighting its potential as a novel approach to combat bacterial infections.

The highest inhibition results were obtained when amoxicillin was combined with each treatment separately; because of that, the MBIC of the amoxicillin alone was at a concentration of 625 μg/mL, while the MBIC values of amoxicillin combined with the different silica NP treatments were less than those concentrations. These results indicated that high-concentration dosage of amoxicillin could be replaced by smaller doses when combined with silica NPs. Hence, fewer toxic side effects would occur.

Amoxicillin was utilized as a positive control to investigate the inhibition of biofilms in vitro. The decision to use this antibiotic was based on its extensive spectrum of activity in combating both Gram-negative and Gram-positive bacteria, making it a common choice for use either alone or in combination. However, the in vivo resistance of *A. baumannii* strains to amoxicillin is notably high, primarily due to their ability to generate biofilms. The growth of these bacterial strains within biofilms necessitates a dosage that is 250 times the antibiotic concentration required to prevent the same strains from growing planktonically in in vitro studies. To account for this, the present study employed amoxicillin in two distinct ways. Firstly, when used as a positive control, a concentration significantly higher than that recorded in previous experiments by [28] was implemented to ensure complete biofilm inhibition. Secondly, when used in conjunction with NPs, amoxicillin was applied in small concentrations to demonstrate the ability of these novel NPs to address resistance caused by biofilm formation.

The MBIC value for amoxicillin was 625 µg/mL in this study, in contrast to other studies, which revealed the MBIC value as being 800 µg/mL [11]. In comparison to the result for amoxicillin, the different silica NPs (pure, composite, alkali, and H_3_PO_4_.SiO_2_) used in this study showed an inhibition effect for the biofilm by reducing absorbance values at various concentrations (from 1.0 mg/mL to 0.125 mg/mL), according to Figure 6. This inhibition activity is caused by the properties of silica NPs, such as their solubility, surface charging, and zeta potential, that interact with proteins or with different elements, hence affecting the bacterial cell. Moreover, cell wall integrity is affected by the electrostatic interaction between negatively charged bacterial cell walls and positively charged NPs [28]. In addition, Lahiri and his colleagues [58] explained the mechanism of the inhibition of QS by the ROS generated by NPs and showed that this caused DNA damage, cell wall destruction, and protein synthesis disruption, ultimately leading to bacterial cell death.

The conclusive results demonstrate the valuable attributes of our innovative nanoparticles, which encompass the safety of human normal cells and exhibit remarkable efficacy even at a minimal dosage, thereby potentially serving as alternatives to traditional antibiotics that biofilm-forming bacteria have developed resistance against.

## 5. Conclusions

The enhancement of bacterial cells’ resistance to antibiotics has emerged as a grave public health concern in the current age. In view of the proliferation of drug-resistant bacteria and the paucity of fresh antibiotics, the present study has proffered innovative strategies to tackle these patient-related predicaments and amplify the potency of traditional antibacterial drugs. This research has substantiated the fact that nanomedicine has a significant role to play in enhancing the effectiveness of existing therapies by bolstering the physical and chemical properties of antibiotics, facilitating the internal integration of biofilms, prolonging the release of antibiotics, enabling targeted delivery to the site of infection, and improving systemic circulation with an associated reduction in side effects. The usage of silica NPs has emerged as a viable alternative solution to address the unwarranted use of wide-spectrum antibiotics and impede the production of biofilm, as evidenced by the results obtained in this study, wherein the sum of the NP treatments administered have demonstrated a pronounced decrease in absorbance values in comparison to the negative control.

The outcomes of this research hold significant value in the domain of biofilm that is created by *A. baumannii*. The study has raised crucial queries such as whether the effects of these treatments are alike in vivo to what is observed in vitro. Additionally, these NPs possess an antiseptic characteristic that may cause biofilm suppression on medical instruments like catheters and central venous catheters (CVCs), thereby reducing the chances of nosocomial infections. Lastly, it remains to be explored whether the variances in bacterial strains influence the efficacy of these human-friendly NPs. All of these inquiries demand further investigation in future studies. Moreover, further research is warranted to explore the underlying mechanisms of these treated NPs’ activity and to optimize the use of these agents in clinical settings.

## Data Availability

The data presented in this study are available on request from the corresponding author.
